# mTICCS and its inter-rater reliability to predict the need for massive transfusion in severely injured patients

**DOI:** 10.1007/s00068-020-01523-w

**Published:** 2020-10-14

**Authors:** Klemens Horst, Philipp Lichte, Felix Bläsius, Christian David Weber, Martin Tonglet, Philipp Kobbe, Nicole Heussen, Frank Hildebrand

**Affiliations:** 1 Department of Trauma and Reconstructive Surgery, University Hospital RWTH Aachen, RWTH Aachen University, Pauwelsstraße 30, 52074 Aachen, Germany; 2 Department of Emergency, Liege University Hospital, Domaine du Sart Tilman, 4000 Liège, Belgium; 3 Department of Medical Statistics, RWTH Aachen University, 52074 Aachen, Germany; 4 Medical School, Sigmund Freud Private University, 1020 Vienna, Austria

**Keywords:** mTICCS, TICCS, Massive transfusion, Shock, Multiple trauma, Polytrauma, Bleeding, Transfusion, Reliability, Inter-rater reliability

## Abstract

**Purpose:**

The modified Trauma-Induced Coagulopathy Clinical Score (mTICCS) presents a new scoring system for the early detection of the need for a massive transfusion (MT). This easily applicable score was validated in a large trauma cohort and proven comparable to more established complex scoring systems. However, the inter-rater reliability of the mTICCS has not yet been investigated.

**Methods:**

Therefore, a dataset of 15 randomly selected and severely injured patients (ISS ≥ 16) derived from the database of a level I trauma centre (2010–2015) was used. Moreover, 15 severely injured subjects that received MT were chosen from the same databank. A web-based survey was sent to medical professionals working in the field of trauma care asking them to evaluate each patient using the mTICCS.

**Results:**

In total, 16 raters (9 residents and 7 specialists) completed the survey. Ratings from 15 medical professionals could be evaluated and led to an ICC of 0.7587 (95% Bootstrap confidence interval (BCI) 0.7149–0.8283). A comparison of working experience specific ICC (*n* = 7 specialists, ICC: 0.7558, BCI: 0.7076–0.8270; *n* = 8 residents, ICC: 0.7634, BCI: 0.7183–0.8335) showed no significant difference between the two groups (*p* = 0.67).

**Conclusion:**

In summary, reliability values need to be considered when making clinical decisions based on scoring systems. Due to its easy applicability and its almost perfect inter-rater reliability, even with non-specialists, the mTICCS might therefore be a useful tool to predict the early need for MT in multiple trauma.

## Introduction

As traumatic haemorrhage and ongoing bleeding are still associated with high mortality rates in patients with multiple injuries, considerable efforts have been made to predict the need for early massive transfusion (MT). Aside from prompt laboratory diagnostics, including standard coagulation parameters and ROTEM^®^, the implementation of MT protocols has been found to improve outcomes [1, 2]. However, the prediction and early identification of patients in need of MT remain difficult [3–5]. Therefore, several scoring systems using imaging, as well as clinical and laboratory parameters, have been introduced and validated [6, 7]. The “Trauma Induced Coagulopathy Clinical Score” (TICCS), which is based on the need for emergency room activation, blood pressure and the presence of significant injuries to different body regions, was introduced in 2014 by Tonglet et al. [8]. A modified version (mTICCS) that automatically considers emergency room activation and does not discriminate between the left and right sides of extremity injury was validated in a population of 33,385 trauma patients in 2017, as its simple applicability predestines it for early prediction of MT soon after trauma [9]. Furthermore, the mTICCS was successfully compared to established and highly sophisticated scores in that field [10]. Based on its three assessment criteria, the mTICCS was found to show no significant difference in the AUC when compared to the AUCs of other established and more sophisticated scores. Due to its simple applicability, diagnosis can be made very early after hospital admission or even in the prehospital setting, and thus the mTICCS might provide a new useful diagnostic tool to detect patients with ongoing bleeding in need of an MT. This might lead to a reduction in the time to therapy initiation [10]. However, the inter-rater reliability of the mTICCS has not been investigated, although reliability values are essential when making clinical decisions based on scoring systems. Thus, the present work aims to evaluate the newly developed and promising mTICCS in regard to its inter-rater reliability.

## Material and methods

### Sample size

Assuming moderate reliability corresponding to an ICC value of 0.5 (null hypothesis) and an expected substantial reliability of 0.7 (alternative hypothesis) in our study, the required number of subjects for a given number of raters to achieve a power of at least 80% at a two-sided significance level of 5% is summarized in the following table (see Table 1). The R Package ‘ICC.Sample.Size’, which is based on Zou (2012), was used to calculate the required number of subjects [11].

**Table 1:** Number of raters and required number of subjects

Number of raters	10	11	12	13	14	15	16
Required number of subjects	29	29	28	28	27	27	27

Based on the assumption that at least 15 clinicians will participate in the survey, and a dropout rate in the evaluation of subjects of at most 5–10% is to be expected, 30 subjects seem sufficient to substantiate the expected effect and were randomly selected from the trauma database for evaluation with the mTICC score.

### Sampling method

A web-based survey (created using www.crowdsignal.org) was sent to 50 European professionals working in the field of trauma care (orthopaedic trauma, general surgery, anaesthesiology and intensive care and emergency medicine) in April 2020. According to the distribution of level 1 to level 3 trauma centres reporting to the TraumaRegister DGU^®^, two thirds of all contacted professionals are working in level 1 centres, while the last third is employed in level 2 or 3 centres. Participants were asked to conduct independently a questionnaire of 30 consecutive case vignettes in which patients sustained a severe injury (see Table 2). All cases were defined as polytrauma when an ISS ≥ 16 points was calculated. Information about trauma severity was based on clinical findings on admission and radiological findings (computertomography). Among these 30 cases, 15 cases (50%) additionally needed a massive transfusion (≥ 10 units of packed red blood cells (pRBC) within 24 h after trauma) due to traumatic haemorrhage.

**Table 2:** Characteristics of the patients

	MT *n* = *15*	No MT *n* = *15*
Age (SD)	33 (14)	41 (20)
ISS (SD)	33 (13)	25 (6)
AIS_head_ ≥ 3 (%)	46.6	46.7
AIS_thorax_ ≥ 3 (%)	66.7	40
AIS_abdomen_ ≥ 3 (%)	53.4	20
AIS_extremities_ ≥ 3 (%)	80	33.3
mTICCS	8 (2)	4 (1)
red blood products (RPB) in mean (SD)	21 (14)	1 (2)

*SD* standard deviation

The survey format and questions were developed and revised by experienced experts at our institution with regard to clarity and overall representativeness of clinical practice. Aside from general data (age, employee status, specialization and experience) participants were asked to rate the probability of MT by the mTICCS, initially developed by Tonglet et al. and validated by Horst et al. [9, 10]. In brief, 16 points can be assigned in three categories: general severity, blood pressure, and the extent of bodily injury (see Table 3).

**Table 3:** mTICCS criteria, *SBP systolic blood pressure

Criteria	Points
General severity	
Admitted to emergency room with trauma team activation	2
Blood pressure	
SBP fell below 90 mmHg at least once	5
SBP always above 90 mmHg	0
Extent of significant injuries for different body regions	
Head and neck	1
Upper extremity (left or right)	1
Lower extremity (left or right)	1
Torso	2
Abdomen	2
Pelvis	2
Total possible score	2–16

### Statistical methods

The intra-class correlation coefficient (ICC) for the assessment of rating reliability was estimated according to Shrout and Fleiss [1979, ICC (2,1)] by means of a linear mixed-effects model with random intercepts for the rater and patient and the variance component as the covariance structure. In addition, a group effect (resident/specialist) was included as the fixed factor for the estimation of employer-status-specific ICCs. Corresponding 95% confidence limits (Cl) were derived as 2.5 and 97.5 percentiles of a bootstrap distribution based on 10,000 samples with replacement from the original data.

For the comparison of ICCs between resident and specialist, a likelihood-ratio test described by Donner and Zou was performed at a significance level of 0.05 [12].

Characteristics of raters, such as age, experience and activation of the trauma team, were described by means and corresponding standard deviations for all raters, as well as being grouped by medical specialists and resident physicians (Table 4). The distributions of sex, specialization and trauma centre level were reported as absolute frequencies; due to the small number of raters, no percentages were provided.

**Table 4:** Characteristics of the raters

Parameter	Resident physicians (*n* = 8)	Specialists (*n* = 7)
Age in years [mean and (SD)]	33 (2)	40 (4)
Sex (male, *n*)	4	6
Specialization (*n*)		
Trauma	6	5
General surgery	1	0
Emergency medicine	0	1
Anaesthesia	0	1
Other	1	0
Experience in years [mean and (SD)]	5 (2)	12 (3)
Trauma centre level (*n*)		
Local trauma centre	0	0
Regional trauma centre	1	1
Supraregional trauma centre	7	4
None	0	1
Other	0	1
Activation of trauma team per year [mean and (SD)]	452 (204)	330 (164)

*SD* standard deviation

All analyses were conducted with SAS version 9.4 (SAS Institute Inc., Cary, NC, USA). The interpretation of the ICC is based on the classification of Landis and Koch (1977). An ICC < 0 reflects ‘poor’ reliability, 0–0.20 ‘slight’, 0.21–0.4 ‘fair’, 0.41–0.60 ‘moderate’, 0.61–0.8 ‘substantial’ and above 0.81 ‘almost perfect’ reliability.

## Results

**Fig. 1:**
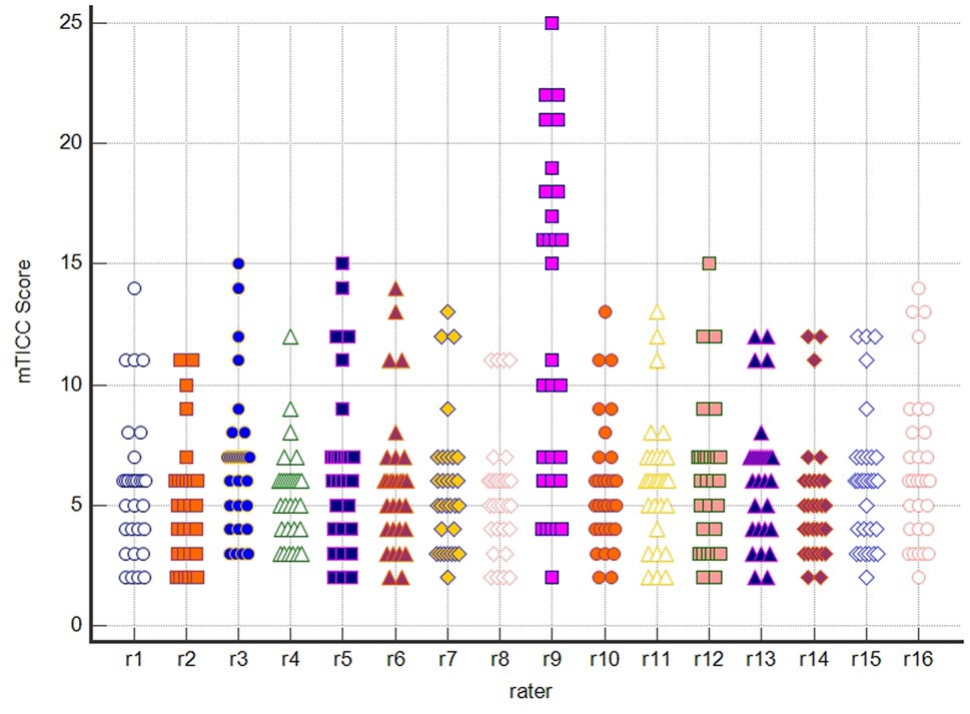
Distribution of the mTICC score for 16 raters on 30 subjects

In total, 30 trauma patients were evaluated by 16 raters (25 did not finish the survey, and 9 made no attempt to participate). All raters were working in urban teaching hospitals. All raters assessed each of the 30 trauma patients independently of each other. One rater assigned scores that were not achievable (see Fig. 1). The corresponding ratings were excluded from the main analysis. The group of 16 raters consisted of 7 medical specialists and 9 resident physicians (8 without the insufficient rating of one rater, see Table 4). Results for the remaining 15 raters are summarized in Table 5.

**Table 5:** Inter-rater reliability (ICC) of all raters and groups by specialist and resident

		95% Confidence limits	
	ICC (2,1)	Lower limit	Upper limit
*k* = 15 rater	0.7587	0.7149	0.8283
*k* = 8 residents	0.7634	0.7183	0.8335
*k* = 7 specialists	0.7558	0.7076	0.8270

ICC < 0 reflects ‘poor’ reliability, 0–0.20 ‘slight’, 0.21–0.4 ‘fair’, 0.41–0.60 ‘moderate’, 0.61–0.8 ‘substantial’, and above 0.81 ‘almost perfect’ reliability

In summary, all 95% CIs of the estimated ICCs ranged from ‘substantial’ to ‘almost perfect’, independent of the educational status (resident or specialist) of the clinicians involved. The likelihood-ratio test did not reveal a significant difference between residents and specialists (*p* = 0.67).

## Discussion

Timely identification of patients in need of MT due to trauma-induced haemorrhage is essential to improve survival [13, 14]. Numerous scores have been developed and validated in the past [7, 10]. Among those, scores including a mixture of clinical parameters, laboratory results, radiological findings and mathematical algorithms presented with the best results [7]. However, the more sophisticated the score, the later an estimation for the potential need of MT can be made in trauma patients with potential traumatic haemorrhage. This is especially true for some relevant variables such as laboratory results and radiological findings, which are not available before the early phase after hospital admission.

As more complex scores have been described to be superior to more compact systems for the prediction of MT, more demanding scores, such as the Trauma-Associated Severe Haemorrhage (TASH) score and the Prince of Wales Hospital (PWH) score, have found wide distribution in clinical practice [15]. Both scores include demographic data, physical variables, laboratory results, injury patterns and sonography. As the TASH presented with the highest area under the curve (AUC 0.889; CI 0.871–0.907) when compared to other established scores like the PWH (AUC 0.860; CI 0.839–0.881), Vandromme (AUC 0.840; CI 0.817–0863), Assessment of Blood Consumption (ABC) (AUC 0.763; CI 0.732–0.794) and the Schreiber and Larsen score (AUC 0.800; CI 0.773–0.828), it is widely accepted as the gold standard in the prediction of MT [7].

The newly developed and easy applicable mTICCS, which is based on emergency room activation, blood pressure and the presence of severe injuries in different body regions, was proven to be a comparably useful tool to the established algorithms [10]. For instance, the AUC of mTICCS (0.776; CI 0.736–0.812) was not significantly different from the AUC of the complex TASH (0.782; CI 0.743–0.819) or PWH score (0.648; CI 0.603–0.691) [10]. Additionally, the present study now proves substantial inter-rater results for the mTICCS. These results were even more remarkable when the score was applied to residents. Thus, the results do not depend on work experience. Literature investigating the established MT scores with regard to their inter-rater reliability are non-existent. However, data from other scoring systems in severely injured patients present with markedly different results with regard to inter-rater reliability. Butcher et al. proved that defining polytrauma by individual subjective perception as well as using scores like ISS or AIS > 3 led to significant disagreement among raters from the same and different institutions [16]. In contrast, using the simple ASA (American Society of Anaesthesiologists) physical classification system showed substantial agreement strength for the reliability of the ASA score among anaesthesiologists (specialists and residents) when evaluating orthopaedic trauma patients [17]. With regard to the highly sophisticated TASH and PWH scores, civilian scenarios with severely injured patients or combat zones, there is an urgent need for more simple tools to stratify patient’s risk for MT [18–20]. Although scores like the Larson, ABC and ET scores are less complex, they still use either laboratory (e.g. base deficit, haemoglobin) or other diagnostic (e.g. x-ray, FAST) variables. However, these variables are probably not available on the scene or hamper timely identification of patients at risk for MT [21]. Even though the requested data might be available in a relatively short time after hospital admission, there will still be a loss of time during data collection and calculation of the scores. Thus, the applicability of these scores as “early” identification tools is questionable and probably explains why these scores are still not used routinely in clinical practice. Moreover, and despite being validated multiple times, studies regarding the inter-rater reliability of the aforementioned scores are not available. Obviously, some of the variables being used for complex scores such as the TASH or PWH score are objectifiable (i.e. sex, laboratory results) and therefore are not susceptible to incorrect scoring. This might explain the missing inter-rater reliability tests for these scores. However, there are still some variables, such as sonography and questions in regard to fracture stability, that are associated with the investigator’s experience and thus provide insecurity in scoring. For instance, focus-assessed sonography in trauma (FAST) is used for many scores aside of the complex ones (i.e. ABC, Emergency Room Transfusion Score (ETS), Traumatic Bleeding Severity Score (TBSS), Massive Transfusion Score (MTS)). However, limitations of FAST exist, especially for particular groups of patients such as children and those with a high injury severity [22]. In this context, Becker et al. reported that the sensitivity and false-negative rate of FAST performed in blunt abdominal trauma patients with a high injury severity score (ISS > 25) are impaired compared to those in patients with an ISS < 25 [23]. Thus, a lower accuracy of FAST due to a higher likelihood of overseen injuries was concluded in more severely injured patients [23]. Accordingly, FAST may not correlate well with the need for an emergent operation [24]. Furthermore, the quality of FAST diagnostics has clearly been shown to depend on the experience of the observer [22, 25]. As a consequence, despite its undisputed value as an extremely useful diagnostic tool in the treatment of trauma patients, the inclusion of FAST as a variable into a scoring system for MT prediction should result in the assessment of the inter-rater reliability of the score.

In addition, the grading of fracture stability plays a crucial role in some scores. Aside from the TASH score, the PWH, the ETS and the TBSS also rely on pelvic fractures as a bleeding source. While an open or dislocated femur fracture, as also used to calculate the TASH score, can be diagnosed easily either by clinical or radiological examination, diagnosis of pelvic stability is more difficult. Against this background, Shlamovitz et al. proved that the presence of either a pelvic deformity or an unstable pelvic ring by physical examination has poor sensitivity for detection of mechanically unstable pelvic fractures in blunt trauma patients [26]. Moreover, Berger-Groch et al. investigated several pelvic scoring systems and found that all classifications reach their maximum reliability with advanced expertise in the surgery of pelvic fractures [27]. Thus, aside FAST another relevant variable used in many established scores offers some susceptibility to grading errors when the diagnosis is not made by experienced medical staff.

## Conclusions

The newly developed mTICCS, based on its three assessment criteria, including activation of the emergency room, blood pressure and the extent of bodily injury, showed substantial results when applied by experts and non-experts alike. Due to its simplicity and good performance, the mTICCS might therefore be seen as a useful diagnostic tool to detect patients with ongoing bleeding in need of an MT. Consequently, the time until life-saving therapy is initiated can be reduced. Prospective follow-up studies, also in a pre-clinical setting, should be carried out to investigate its clinical value.

### Strengths and limitations

The present analysis was based on a questionnaire including real patient vignettes from severely injured patients. All included variables in the investigated scoring system could be assessed from the hospital information system. In addition, detailed data on transfusion practices and the use of blood products were available. In contrast, data presented in the current study were collected from a retrospective database with information collected after hospital admission. Thus, information about pre-hospital applications of haemostatic agents with a potential influence on the amount of administered pRBCs were not available and might have caused some bias in the calculation of mTICCS. Furthermore, combining anatomical and physiological parameters have shown to give a more precise information about trauma severity. However, not all of these parameters were available. Taking these shortcomings into account, prospective studies of the presented score in a pre-clinical setting should be planned.

## Data Availability

Data are stored at our institution and can be assessed by researchers who meet the criteria for access to confidential data.
